# An initial study on the agreement of body temperatures measured by infrared cameras and oral thermometry

**DOI:** 10.1038/s41598-021-91361-6

**Published:** 2021-06-07

**Authors:** Scott Adams, Tracey Bucknall, Abbas Kouzani

**Affiliations:** 1 School of Engineering, Deakin University, Geelong, Australia; 2 School of Nursing and Midwifery, Deakin University, Geelong, Australia; 3 Nursing Services, Alfred Health, Melbourne, Australia; 4 Centre for Quality and Patient Safety-Alfred Health Partnership, Institute for Health Transformation, Melbourne, Australia

**Keywords:** Population screening, Biomedical engineering, Fever

## Abstract

The COVID-19 pandemic has led to the rapid adoption and rollout of thermal camera-based Infrared Thermography (IRT) systems for fever detection. These systems use facial infrared emissions to detect individuals exhibiting an elevated core-body temperature, which is present in many symptomatic presentations of Severe Acute Respiratory Syndrome Coronavirus 2 (SARS-CoV-2). Despite the rollout of these systems, there is little independent research supporting their efficacy. The primary objective of this study was to assess the precision and accuracy of IRT screening solutions in a real-world scenario. The method used was a single-centre, observational study investigating the agreement of three IRT systems compared to digital oral thermometer measurements of body temperature. Over 5 days, 107 measurements were taken from individuals wearing facial masks. During each entry, two measurements of the subject’s body temperature were made from each system to allow for the evaluation of the measurement precision, followed by an oral thermometer measurement. Each participant also answered a short demographic survey. This study found that the precision of the IRT systems was wider than 0.3 °C claimed accuracy of two of the systems. This study also found that the IRT measurements were only weakly correlated to those of the oral temperature. Additionally, it was found that demographic characteristics (age, gender, and mask-type) impacted the measurement error. This study indicates that using IRT systems in front-line scenarios poses a potential risk, where a lack of measurement accuracy could possibly allow febrile individuals to pass through undetected. Further research is required into methods which could increase accuracy and improve the techniques viability.

## Introduction

Core body temperature is one of the four key vital signs, which is regularly assessed by healthcare settings, alongside respiration rate, blood pressure and heart rate^1^. In an in-patient setting, core body temperature can be assessed from different body locations using oral, rectal, tympanic or temporal artery thermometers, or even through urinary or pulmonary artery catheters with in-built temperature sensors^2^. The accuracy, precision, advantages and disadvantages of these temperature measurement devices in clinical settings has been well established^2–4^.

In addition, in modern medical practice, every device must be assessed against national and international regulations. To ensure that a device meets appropriate levels of quality, accuracy and safety, strict medical equipment certification standards must be met by the device prior to its use in a clinical setting. The bodies administering these standards for medical devices include the Therapeutic Goods Authority in Australia, the Food and Drug Administration in the USA and the Medicines and Healthcare products Regulatory Agency in the UK^5^. The use of evidence-based assessment to evaluate technologies for use in a hospital setting is a common and critical part of modern healthcare, ensuring patient safety and assisting in the delivery of high-quality care^6^.

The COVID-19 pandemic has led to an unprecedented adoption and rollout of new fever detection technologies^7^. As fever is present in a significant proportion of symptomatic SARS-CoV-2 cases, the goal of the screening is to identify individuals exhibiting an elevated temperature, isolate them, and refer them for a more comprehensive assessment to a health practitioner^8^. Currently, fever screening technologies are typically installed in high-traffic areas, such as train-stations or airports, and also at the entrance of high-risk sites, such as hospitals, where the consequences of an outbreak could be catastrophic. Many of the deployed fever screening solutions have not yet been assessed by regulatory agencies.

The ideal screening technology must be accurate, rapid, widely available, and operate in a way that keeps both the test administrator and subject safe from viral transmission. In addition, an ideal solution would operate without consumables to deal with global supply chain shortages of critical resources, such as consumables, personal protective equipment (PPE) and medical devices, which have been experienced during the COVID-19 pandemic^9^. When compared to this ideal screening technology, it is clear that traditional measurement techniques have a variety of limitations which restrict them from being highly suited for use as mass-screening tools. Traditional hospital-grade, contact-based measurement techniques all require close proximity between the test administrator and the subject, and some methods are too invasive, too slow, or too expensive to be widely used. This has led to the increased adoption of infrared thermal detection systems for fever screening applications.

Infrared thermal detection systems operate through the measurement of thermal radiation emitted in the infra-red wavelengths, of the electromagnetic spectrum^10^. The thermal radiation is converted through a transducer to an electrical signal which can be interrogated and measured on-board of the device. In fever screening applications, detection systems fall into two main categories: handheld Non-Contact Infrared Thermometry (NCIT) devices, and Infrared Thermography (IRT) systems. These systems meet many of the criteria for a successful mass screening system, they are non-contact, require no consumables, are rapid, and in the case of IRT systems the operator can be physically distanced from the subject. While the use of NCIT devices has been explored in a hospital setting through a number of clinical trials and research articles, and that many of NCIT devices have achieved appropriate medical device approvals, these are not yet established for most IRT systems^11–13^.

Despite the wide-spread use of IRT systems, there remains limited independent evidence demonstrating their efficacy and accuracy when measuring body temperature for fever screening. Some clinical trials have been conducted, but the results have been mixed, and there is a lack of consensus in the literature on the effectiveness of IRT systems. A 2015 study in Singapore found that one system was able to achieve a high level of sensitivity and specificity (89.7% and 92%, respectively)^14^, and a similar result found from a study conducted in the USA in 2010, reporting a sensitivity of 91.0% and specificity of 86.0%. However, these results were not found to be broadly repeatable, as revealed by three experiments performed in Hong-Kong and New Zealand between 2011 and 2013^15–17^. The most recent of these studies only reported a maximum sensitivity and specificity of 64% and 86%, respectively, when measurements were taken in a comparative manner^15^. International standards such as the ISO/TR 13154:2017^18^ which are used to explain and outline current best-practice approaches suggest a number of considerations to be taken into account (e.g. measurement location, number of subjects who can be measured simultaneously, and recommended distance to subject) when performing fever screening. The existing studies did not report that any of the systems which were installed according to these standards. This is likely due to the fact that the finalised version of the ISO/TR 13154:2017 standard was released after the studies were conducted.

As such, this study, conducted in Australia during the COVID-19 pandemic, evaluates the precision and accuracy of three different types of IRT system for human temperature measurements when installed in a real-world scenario with a mask-wearing population. This was performed to determine their efficacy as screening systems, when compared with a certified benchmark temperature measurement device commonly used in hospitals.

## Method

### Study design

The investigation was designed as a single-centre observational study comparing the accuracy of three IRT systems to core-temperature measurements taken using certified oral thermometers. Additionally, the precision of each IRT system was determined through repeat measurements.

### Setting

The study was conducted at a University during a 5-day period in August 2020 from 9 a.m. to 5 p.m.

### Sampling and eligibility criteria

The study was performed on a community sample and used a convenience method for participant recruitment. The study aims, participation requirements, and methods of consent and requirements of participation were provided in an email to staff and students in the building. Verbal consent was gathered on the day. These measures were performed to ensure that social distancing guidelines were able to be maintained during the study. Every employee and student who attended the building during the study period was invited to participate. Due to the nature of the facility, the participants were all over 18 years of age.

### Ethical considerations

This study was approved by the Deakin Human Ethics Advisory Group (Health) (approval number: HEAG-H 154-2020) at Deakin University (Australia). All methods were performed in accordance with the recommendations of the Ethics Advisory Group as well as the relevant guidelines and regulations. Informed consent was gathered from every participant, prior to their inclusion in the study and all data was anonymised before being stored in a secure double identifier password protected REDCap (Research Electronic Data Capture) database administered by Deakin University with access limited to the study investigators.

### IRT systems

Three IRT systems were selected for use which represented three of the main types of systems that are being sold in the Australian market. These were as follows:

- System 1—A dual-camera system with a 40 °C external reference temperature device (blackbody), advertised to be able to measure up to 30 subjects simultaneously while performing facial recognition tasks.
- System 2—A single camera system with laser-assisted autofocus which operates without a blackbody.
- System 3—A single camera system with a 35 °C blackbody which is deployed in-line with the guidance provided in the ISO/TR 13154:2017 technical standard (apart from the guidance on masks)^18^.

The specifications for each system are included in Table 1:

**Table 1:** Specifications of the IRT systems used in the study.

	System 1	System 2	System 3
Camera pixels	400 × 300	464 × 348	640 × 480
Thermal sensitivity	≤ 40 mK	< 40 mK	< 50 mK
Stated accuracy	± 0.3 °C	± 0.3 °C	Not stated
Dual cameras	Yes	No	No
Blackbody used (temperature)	Yes (40 °C ± 0.1 °C)	No (N/A)	Yes (35 °C ± 0.1 °C)
Measurement location	Whole face	Inner canthus	Inner canthus
Data reporting	Corrected	Raw	Raw
Number of subjects in simultaneous measurement	30	1	1
Required a sightboard	No	Yes	No

### Experimental setup

Each of the three IRT systems were loaned to the researchers by the manufacturing companies for the purpose of conducting this experiment. To ensure the independence of this trial, each company provided the equipment free of charge and signed a research services waiver giving the researchers the right to publish the results without commercial input or oversight. The IRT systems were all setup according to the manufacturer’s directions. The researchers were provided training on the assembly, deployment and operation of the IRT systems to ensure correct operation. Each manufacturer confirmed the configurations of the systems, the setup environment and the experimental methodology prior to the beginning of the trial through video conference but were otherwise uninvolved in the experiment.

**Figure 1:**
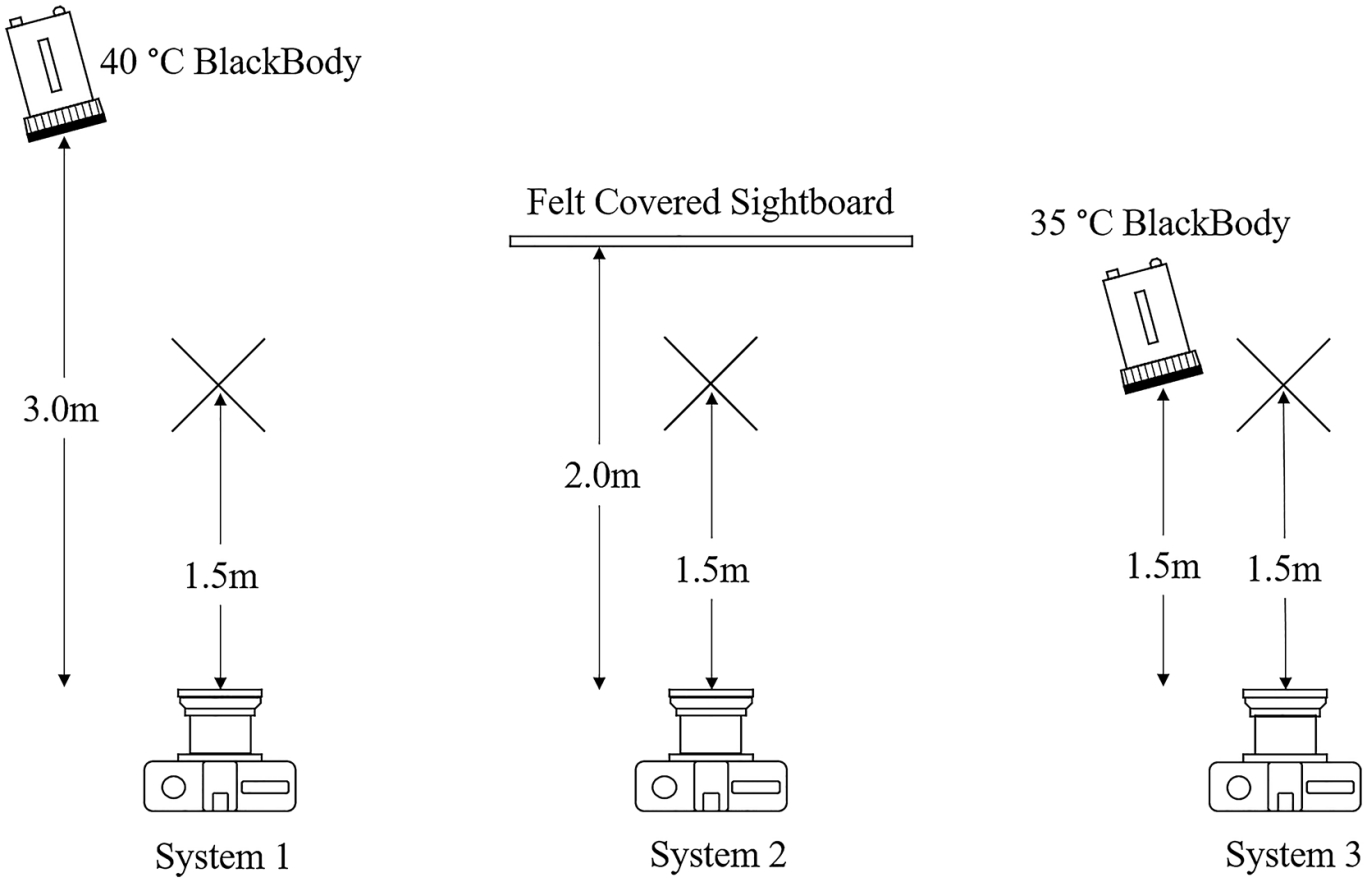
Experimental setup of the three IRT systems. In each case, the location of the subject being measured is indicated by a cross.

The setup conditions of the three IRT systems are visualised in Fig. 1.

The conditions of the experimental environment were as follows:

- The area was not directly under any active Heating, Ventilating, and Air Conditioning (HVAC) system.
- The room in which the trial was located was temperature and humidity controlled.
- No lights or thermal radiation sources were directly in view of any of the cameras, and the cameras were not pointed at any reflective surfaces.
- Each IRT system was allowed 30 min of temperature stabilization each day prior to the first measurement being taken.
- The room had no direct entry from the outside, each doorway had an airgap which had to be traversed prior to entering the experimental area.
- Systems 2 and 3 were focussed at the beginning of the trial, and focus was checked four times per day (the systems remained in focus throughout the trial). The manufacturer of System 1 indicated that manual focussing was not required.
- Systems 2 and 3 were directly in-line with the subject’s face. The manufacturer of System 1 indicated that this was not necessary.
- TV Screens were setup for Systems 2 and 3 so that subjects could orient themselves within the camera targeting area. System 1 used a web-based application and a laptop to display the data.

### Experimental procedure

Every step of this experiment was strictly conducted according to socially distancing guidelines between the researcher and the subject.

As a potential participant entered the building, they were approached by the researcher who confirmed their knowledge of the study (information which had been provided through email) and asked for their verbal consent. If the answer was in the affirmative, the participant then verbally completed a questionnaire of demographic data (age, gender, and mask-type), and also was queried as to their current health status, in particular:

- “Have you experienced any fever symptoms in the last 24 h?”
- “If yes, did you take any medications to treat your fever in the last 4 h?”
- “Have you experienced any symptoms in the last 24 h of sore throat, cough, runny nose, loss of taste or smell?”
- Have you had a hot or cold drink in the last 30 min?

This data was entered into a case report form for each individual in a secure REDCap (Research Electronic Data Capture) database.

The participant was then questioned to if they had been outside the building in the past 5 min, if the answer was in the affirmative, they were asked to wait for 5 min to acclimatise. The purpose of this was to avoid readings from being impacted by the exterior environment. The participants were also asked to remove hats or glasses to ensure accurate readings.

All participants were wearing masks during the trial, apart from when using the oral thermometers, as during the SARS-CoV-2 pandemic in Victoria, Australia, there was a state-wide mandate that all individuals must wear masks when outside their own homes, this means that any screening technology used during this time would be required to operate with masked participants^19^.

Each participant was then asked to spend 5 s standing in front of each IRT system facing directly into each camera at a distance of 1.5 m (indicated by a mark on the floor). This was then repeated, to give two readings for each time an individual participated in the experiment. At the conclusion of these measurements (6 in total), the participant was provided with a DT-01B oral thermometer (Measurement accuracy: ± 0.1 °C [35.5–42.0 °C]) and requested to stand in an isolated location (indicated by a mark on the floor), where they proceeded to take an oral thermometer reading. The two readings from each IRT system and the single oral thermometer reading were then entered into the secure REDCap database case report form.

### Analysis

The RStudio integrated development environment for R (version 1.3.1056, R version 4.0.2) was used for the statistical analysis, and for all tests, a 0.05 level of significance was used. The frequency distribution (reported as mean and standard deviation), was calculated for the participant’s age, the number and percentage distribution of the participant’s gender, and mask-type were also calculated.

An investigation into the measurement precision was then performed to analyse the difference between the first and second measurements of each IRT system and to determine if the claimed 0.3 °C of accuracy was observed in a multi-measurement precision test. This precision error was also reported as a boxplot to observe the distribution of the quartiles as well as to identify outliers. The frequency distribution of the precision error was then calculated and reported as mean and standard deviation.

The measurements of the IRT systems were then compared against the reading of the oral thermometer, and the mean and standard deviation were calculated. The Pearson's correlation coefficient (ρ) was also calculated to determine the correlation between the measurements. The thermal camera measurements, in comparison to the oral thermometer readings were then fitted to a linear model, and the coefficient of determination for each system was calculated. The error of each measurement for the three systems was then calculated. Finally, the accuracy was assessed in relation to each of the demographic attributes (age, gender, mask type), the mean and standard deviation of the error was calculated, and tests of statistical significance between the systems were calculated using Welch’s t-test (as the sample sizes were unequal^20^).

## Results

### Participant characteristics

Over the 5-day period of the study, a total of 107 measurements were taken from the participating subjects within the building. Each participant was measured twice by each system in the manner described in the Experimental Procedure, giving a total number of thermography measurements as 214. As individuals were able to take part in the study on subsequent days, the number of unique participants was seventy-one. Table 2 details the demographic attributes of the study participants. All participants wore face masks as mandated by the Victorian Department of Health and Humans Services^19^.

**Table 2:** Demographic attributes of participants.

Characteristics of participants, N = 107		
	Mean	SD
Age (year)	35.66	10.09
Self-reported fever	0	0
Other symptoms of COVID-19	0	0
**Gender**	**n**	**%**
Male	77	71.96
Female	30	28.04
**Mask type**	**n**	**%**
Thin	56	52.34
Thick	51	47.66

In this study, no participants reported having a fever in the past 24 h, or experiencing other COVID-19 related symptoms (such as sore throat, cough, runny nose etc.) which is due to a level of selection-bias, as these symptoms would have precluded their entrance to the campus.

### IRT system precision test

**Figure 2:**
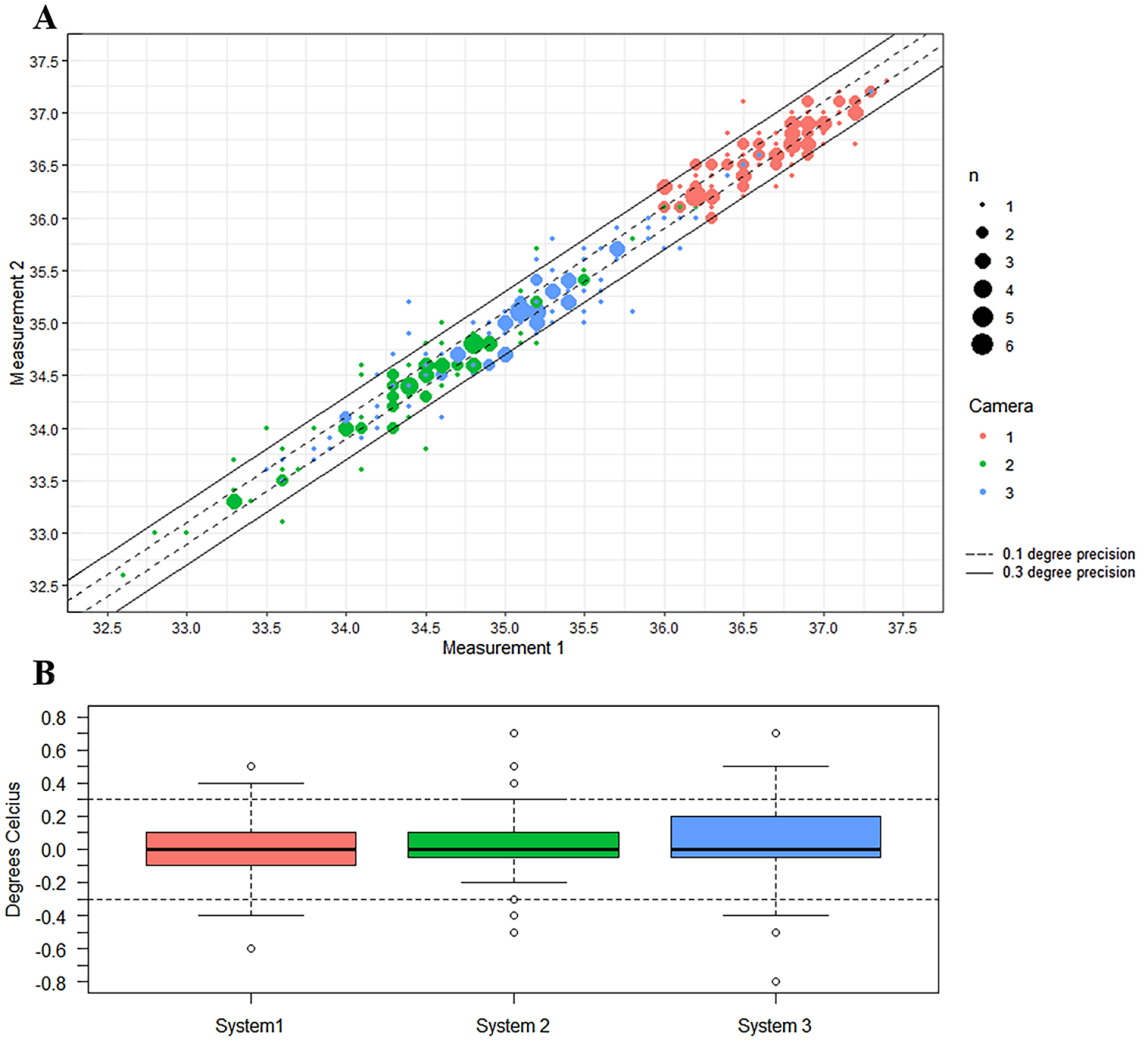
Repeated measurements precision test results. (**A**) A bubble-plot which displays the first measurement against the second. The dotted lines is the range in which a system with 0.1 °C of precision would fit, the continuous lines are the range in which a system with 0.3 °C of precision would fit. (**B**) A boxplot of the difference between the first and second measurement clearly displaying the quartile ranges and the outliers from each system.

The first test performed on the IRT systems was to determine if the systems were able to achieve a precision of within ± 0.3 °C through a repeated measurement precision test, the results are shown in Fig. 2 and Table 3. As each participant in the study was measured twice by each system within 30 s, the ideal system would have had a temperature difference of 0 °C between the two measurements. From our results it was clear that none of the systems achieved a precision within ± 0.3 °C. The mean and standard deviation of precision error for each system were as follows: System 1: 0.024 ± 0.183 °C, System 2: 0.019 ± 0.194 °C, and System 3: 0.044 ± 0.214 °C. The mean errors were low in this case as all systems experienced both positive and negative precision errors which caused error cancellation. Using the 2-standard deviation confidence interval, the systems were found to have precisions of: ± 0.34 °C for System 1, ± 0.37 °C for System 2 and ± 0.38 °C for System 3. From the box-plot in Fig. 2B, it can be clearly observed that System 2 was the system with the most measurements within ± 0.3 °C measurement precision, however it still recorded a number of outliers.

**Table 3:** The data from each IRT system, and the oral thermometer. The correlation coefficient is for each IRT system against the oral thermometer.

System	Mean (SD) °C	Mean difference °C	Correlation coefficient (ρ)
Oral thermometer	36.37 (0.452)	–	–
System 1	36.62 (0.338)	0.255	0.333
System 2	34.47 (0.685)	− 1.902	0.407
System 3	35.06 (0.671)	− 1.311	0.491

### IRT system accuracy and correlation test

The second test compared the IRT system readings against the oral thermometer to determine how correlated the readings from each system were to core body temperature. While not required to be exact, an accurate system for detection of elevated body temperature should have a strong degree of correlation with the oral thermometry readings, with an increase in core temperature resulting in an increased IRT measurement. The results of this test were tabulated and are displayed in Table 3. The oral thermometer measurements were similar to those found in previous studies^3,4^. Firstly, there were significant differences found between the IRT systems and the oral thermometer measurements with the mean difference in System 1 being 0.26 °C, System 2 being − 1.90 °C, and System 3 being − 1.31 °C. In addition, the correlation coefficients calculated were weak (< 0.5), with System 3 being the most correlated to the oral thermometer results (ρ = 0.49) and System 1 being the least (ρ = 0.33).

**Figure 3:**
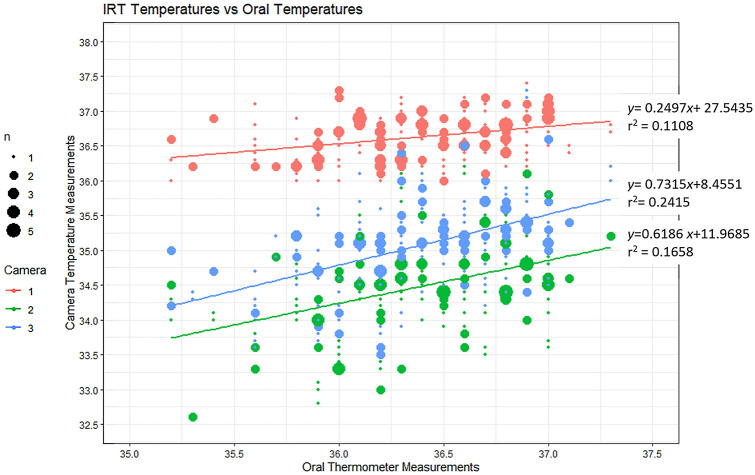
Bubble-plot of the IRT measurements vs the oral thermometer measurements.

A linear model was developed for each of the systems relative to the oral thermometer results and is displayed as a bubble-plot in Fig. 3. Each of the systems had a generally increasing trend in their reported temperature readings as the oral thermometer readings increased. However, the results from this analysis agree with the results in Table 3 with the three IRT systems only presenting weak coefficient of determination’s (r^2^), with the strongest being System 3 (r^2^ = 0.24) and the weakest being System 1 (r^2^ = 0.11).

### Participant characteristics and IRT system error

In addition to examining the participants in aggregate, a set of analyses was also performed to determine if any of the recorded demographic attributes had an impact on the measurement error experienced by the IRT systems compared to the oral thermometer. For each of the recorded characteristics (age, gender, and mask type), the mean and standard deviation of the error and p-values were calculated, using Welch’s t-test (as the sample sizes are unequal^20^). These results were tabulated and are displayed in Table 4. From the data, it can be seen that the IRT systems errors were impacted by the demographic factors, however, each of the systems was not impacted by the same demographic factors. System 1 was impacted by the age of the subject as well as by the mask type. System 2 and 3 were impacted by the participants gender.

**Table 4:** Results of examining the error of each IRT system in relation to each of the recorded characteristics. Factors which have a p-value of < 0.05 are shaded. N = 214.

	n	System 1		System 2		System 3	
		Mean (SD) °C	p-value	Mean (SD) °C	p-value	Mean (SD) °C	p-value
**Subjects age**^a^							
< 40	150	0.192 (0.415)	< 0.05	− 1.874 (0.662)	0.86	− 1.277 (0.594)	0.31
≥ 40	44	0.395 (0.525)		− 1.855 (0.627)		− 1.389 (0.643)	
**Gender**							
Male	154	0.273 (0.480)	0.35	− 1.762 (0.608)	< 0.05	− 1.211 (0.569)	< 0.05
Female	60	0.210 (0.424)		− 2.260 (0.615)		− 1.570 (0.595)	
**Mask type**							
Surgical mask	112	0.339 (0.456)	< 0.05	− 1.884 (0.693)	0.67	− 1.271 (0.609)	0.30
Thick mask	102	0.163 (0.459)		− 1.922 (0.599)		− 1.356 (0.584)	

^a^Subjects were given the option to not give an age, so 20 measurements were not included in the age analysis.

### Validation of System 2 against external temperature reference devices

System 2 was checked against two separate blackbody reference devices (35 ± 0.1 °C and 40 ± 0.1 °C) and was found to report the correct temperature within 0.1 °C, demonstrating its high degree of measurement accuracy.

## Discussion

IRT systems are being installed in a wide variety of locations worldwide in response to the COVID-19 pandemic. Yet, there is limited evidence available in the literature reporting on their accuracy or efficacy in these application spaces, in particular on how they correlate to core-body temperature measurements. In order to address this research gap, the current study investigated the use of three different IRT systems from different manufacturers in a real-world setting, with a community mask-wearing population.

Firstly, the precision of each of the three systems was determined through repeated measurements taken a short time apart (< 30 s). Each of the systems were found to have a precision wider than the 0.3 °C accuracy claimed by two of the systems. Secondly, in regard to measurement accuracy, our results suggest that the IRT systems each experience a deviation from the core temperature measurements. Thirdly, this study has shown that in our sample, the IRT systems measurements only have a weak correlation to the oral temperature measurements, with System 3 having the highest ρ and r^2^ with values of 0.49 and 0.24, respectively. Fourthly, the participant characteristics were associated with changes in the error between oral measurements and IRT systems measurements, however, these were not consistent across all three IRT systems.

Some existing studies have found IRT systems to be sensitive and specific in assessing the febrile status of subjects^14,21^. These studies were also conducted with participants > 18 years of age and with multiple IRT systems. However, neither of these studies were conducted with a mask-wearing population, and nor with IRT systems installed at the first stage of entry to a building as in our study, which is a common use-case for these systems in hospitals and workplaces in 2020. In addition, the study by Nguyen et al.^21^ which found the IRT systems to be sensitive and specific, was performed on individuals after they had been registered in the emergency department, which may have allowed for an extended period of acclimatisation time when compared to a regular building entry.

Our study is the only recent study reporting on repeated measurement precision of the IRT systems, this is a significant result as it describes the repeatability of the system when measuring the same person multiple times. From our results it seems unlikely that a 0.3 °C level of accuracy would be achievable with such a wide measurement precision in each of the systems. Indeed, all 3 of the systems reported measurements with differences of > 0.5 °C on the same individuals only 30 s apart.

The research conducted by Ghassemi et al.^22^, and the ISO/TR 13154:2017 standard suggest that using an external reference device (blackbody) increases the accuracy of the measurements. Our study results show that this is a good recommendation; System 3, which used a blackbody reference, had a mean difference 0.59 °C closer to the core temperature measurement than System 2, which used a similar camera without a blackbody. In addition, System 3 was found to have a greater correlation to core temperature than System 2, with their respective correlation coefficients being 0.491 and 0.407.

The current ISO standards also recommend that measurements should be taken from the inner canthus of the eye rather than the forehead or general facial measurements^18,22^. Our study found that System 1 which was not specifically taking measurements from the inner canthus in fact reported measurements which were closest to the core body temperature measurements (mean difference increase of 0.26 °C). This was an unexpected result, as the literature suggests that the facial skin temperature is generally expected to be lower than the core-body temperature^16,23^. This suggests that System 1 is employing a correction algorithm on the measurement results, which may shift the measurements into a more “acceptable” range. Additionally, the measurements from System 1, which were not taking measurements from the inner canthus were found to have the smallest coefficient of determination of any of the systems to the core body temperature (r^2^ = 0.11). This suggests that the use of the algorithm does not significantly improve the efficacy of whole-face temperature measurement, and that the current guidance is correct in recommending measurements be taken from the inner-canthus region over a general facial measurement.

The correlation found in our study (maximum being ρ = 0.49) is generally in agreement with the existing literature, which reported values between 0.3 and 0.5 for well-functioning systems^15,21,24^. However, the study by Chan et al.^15^ found that the correlation is higher among febrile populations with core-temperatures ≥ 38 °C, which we were not able to include in our study, so the correlation may have been improved with a population including a large cohort of febrile individuals.

The experiment involving the validation of System 2 against external temperature reference devices (blackbodies) demonstrated that this system is capable of measuring emitted thermal radiation with a high degree of accuracy in the experimental environment. When measuring these near-ideal sources, System 2 reported a measurement value within 0.1 °C of the expected temperature, which is within the margin of error of the reference device. This suggests that the measurement error observed in experiments with human subjects is likely due to the physiological link between core-temperature and facial temperature, rather than inherent technological error.

Existing research into normal body temperatures has found that the normothermic difference between different demographics (age, gender, etc.) is smaller than the error range of our measurement devices (0.3 °C)^4^, thus a study into the temperature differences of demographics would be of limited use. Thus, this work investigated how these demographic factors impact the accuracy of the measurements taken when using IRT measurement systems. Our study found that demographic characteristics had a significant impact on the measurement error of the systems, however, this was not consistent across the three IRT systems. System 1 which measured the whole face temperature exhibited an increased error on subjects ≥ 40 years of age, and those who were wearing thinner masks. Systems 2 and 3 which measured the inner canthus of the eye had an increase in error on subjects who identified as female. This is in agreement with existing literature which suggests that gender and age are factors which impact measurement accuracy of infrared thermography systems^15,21^.

To the authors’ knowledge, this is the first IRT study which has reported face-mask type as a demographic factor and investigated its impact on measurement error. Our earlier study using NCIT devices in a hospital setting found that demographic factors (age, skin tone and gender) also impacted measurement results, so it appears that there is scope for future research to determine their precise impact on the performance of infrared measurement systems in fever-screening scenarios^12^. Finally, we do not believe that the use of masks had a significant impact on the effectiveness and efficacy of the measurements being taken. As the systems were not targeting regions of the face covered by the mask it is unlikely that the inclusion of a mask altered these temperatures, particularly the systems that were measuring the inner canthus region. From observing the systems during operation it was clear that each system was able to quickly and correctly identify the facial location to be measured, indicating that the masks did not make an impact on the facial positioning algorithms being used. This could be confirmed through a specific research study.

### Strengths and limitations

To the authors’ knowledge, this the first study of this type which has been conducted on a mask-wearing population, which was a mandated intervention in Victoria, Australia during parts of the COVID-19 pandemic. The limitations of the study were as follows: this was a convenience selected community sample, with no febrile subjects, there were no subjects > 65, the duration of the camera loan agreements and facility agreements dictated a one week timeframe which restricted the sample size. Additionally, the variations in height of individuals impacted the measurement process as some subjects had to bend, lean, or use chairs in order to be in focus in the measurement. Also, this study had a larger population of male (71.96%) than female (28.04%) participants, this may impact the translation of the results. The inclusion of an NCIT device could have allowed for the exploration of the source of the measurement error, and finally the lack of febrile individuals made it impossible to assess the sensitivity and specificity for individual febrile detection.

### Future work

There is a clear need to expand this study into a setting with more febrile individuals to allow for the assessment of sensitivity and specificity of fever detection, however, the low correlation values found between each of the measurement sources raises doubt to the efficacy of these systems at detecting individuals with low-grade fevers (37.5–38 °C). Also, there is clearly scope for future research into investigating the impact of camera characteristics on measurement results. This would involve creating a study where multiple cameras with varying characteristics from multiple manufacturers would be tested in the same setup conditions to determine the impact of the technical characteristics on the measurement results. Another avenue of investigation for future research would be a quantitative determination of the impact of masks on IRT system measurements. A study in which each participant is measured both masked and un-masked and the associated results compared, would be a great addition to the literature and allow for a quantitative determination of the impact of masks on IRT measurements. In addition, there is clear scope to perform further investigations into the impact of age and gender on the measurement results. Finally, there is clearly an interesting avenue of investigation in relation to improving the precision of these measurement techniques for mass screening (Supplementary Information).

## Conclusion

This paper presented the first study on assessing the capabilities of IRT systems in a face-masked population in a real-world mass screening scenario. This system was tested outside of a hospital setting, at the entrance to a research facility within a building, mimicking the installation scenario of many currently operating IRT systems. Our results show that using the systems as a front-line intervention for fever-screening poses a potential risk, where the lack of measurement repeatability could negatively impact sensitivity and specificity, possibly allowing febrile individuals to pass through undetected. Although these systems are currently seeing widespread use due to the COVID-19 pandemic, our results show that there is still further research required to improve their precision and accuracy so that users can be confident in their operation. There remains an opportunity for new technology to meet this gap.

## Supplementary Information

Supplementary Information.
